# Effect of Copper Nanoparticles in the Diet of WKY and SHR Rats on the Redox Profile and Histology of the Heart, Liver, Kidney, and Small Intestine

**DOI:** 10.3390/antiox11050910

**Published:** 2022-05-06

**Authors:** Ewelina Cholewińska, Jerzy Juśkiewicz, Michał Majewski, Radosław Smagieł, Piotr Listos, Bartosz Fotschki, Irena Godycka-Kłos, Katarzyna Ognik

**Affiliations:** 1 Department of Biochemistry and Toxicology, Faculty of Animal Sciences and Bioeconomy, University of Life Sciences, 20950 Lublin, Poland; ewelina.cholewinska@up.lublin.pl (E.C.); radoslaw.smagiel@up.lublin.pl (R.S.); 2 Division of Food Science, Institute of Animal Reproduction and Food Research, Polish Academy of Sciences, 10748 Olsztyn, Poland; b.fotschki@pan.olsztyn.pl (B.F.); i.godycka-klos@pan.olsztyn.pl (I.G.-K.); 3 Department Pharmacology and Toxicology, University of Warmia and Mazury, 10082 Olsztyn, Poland; michal.majewski@uwm.edu.pl; 4 Department of Pathomorphology and Forensic Medicine, Faculty of Veterinary Medicine, University of Life Sciences in Lublin, ul. Głęboka 30, 20612 Lublin, Poland; piotr.listos@up.lublin.pl

**Keywords:** hypertension, copper nanoparticles, rat, redox status, tissue, histology

## Abstract

The aim of this experiment was to test the effect of the partial or complete replacement of traditional CuCO_3_ in the diet of rats with copper nanoparticles (CuNPs) on the biochemical parameters, redox status, and histomorphometry of their tissues. Normotensive male Wistar–Kyoto rats (WKY) were allocated to three groups. Three analogous groups of spontaneously hypertensive rats (SHR) were also formed. The WKY and SHR rats received copper in a standard daily dose—6.5 mg/kg CuCO_3_ or CuNPs (100% replacement) or 3.25 mg/kg CuCO_3_ plus 3.25 mg/kg CuNPs (50% replacement)—for 8 weeks. Next, blood, heart, small intestine, liver, and kidney samples were collected. The activity of alanine aminotransferase, aspartate aminotransferase, creatine kinase, and gamma-glutamyl transferase and the content of creatinine and urea acid were measured in the plasma. The collected tissues were subjected to a histological evaluation, and redox status parameters (catalase and superoxide dismutase activity, malondialdehyde and glutathione content) were determined. The replacement of CuCO_3_ with CuNPs in the diet may exacerbate the negative changes induced by hypertension in the heart, liver, and intestines. However, it seems that it is only in the case of the liver where the observed changes may be due to an increase in oxidative reactions resulting from the inclusion of CuNPs.

## 1. Introduction

Hypertension is one of the most common chronic diseases of the modern world and significantly increases the risk of premature death [1]. Hypertension is believed to result from a combination of factors, including genetic predisposition, a high-calorie or high-salt diet, and ageing processes in the body [2]. It is often called ‘the silent killer’ because in many cases, it develops without symptoms for a very long time, leading to serious damage to internal organs [1,2,3]. Elevated blood pressure has been shown to be a significant risk factor in the development of conditions such as chronic heart disease, stroke, coronary disease, ischaemic heart disease, heart failure, atherosclerotic changes in blood vessels, kidney dysfunction, retinal bleeding, retinopathy, and visual impairment [2,4,5] This means that it is the heart, arteries, kidneys, and eyes that are most susceptible to damage caused by haemodynamic overload resulting from high blood pressure [1]. However, the liver and even the intestines are also increasingly mentioned as target organs of hypertension [3]. The available literature indicates that oxidative stress plays a key role in the pathogenesis of hypertension [4,6], as nitric oxide (NO) released by endothelial cells plays an important role in artery relaxation processes. This compound is rapidly degraded due to the activity of superoxide anion, a reactive oxygen species. Superoxide anion not only limits the bioavailability of NO, but also directly affects blood vessels, causing their constriction. A reduced NO level and the excessive contraction of blood vessels induced by the presence of superoxide anion leads to the development of hypertension. This in turn leads to a further increase in oxidative stress and thus to damage to internal organs and metabolic disorders [4,6]. There are also reports indicating that angiotensin II, which is part of the renin-angiotensin-aldosterone (RAA) system that regulates the volume of blood circulating in the body, may stimulate nonphagocytic NADPH oxidase to produce excessive amounts of free radicals—hydrogen peroxide, superoxide, and peroxynitrite. This leads to blood vessel damage and kidney dysfunction [7,8]. The existence of a link between oxidative stress and elevated blood pressure is supported by research from Pedro-Botet et al. [9], who demonstrated the reduced activity of the antioxidant enzymes superoxide dismutase and glutathione peroxidase, accompanied by the increased production of hydrogen peroxide and lipid hydroperoxides, in patients with hypertension.

It is believed that the effects of increased oxidative stress accompanying hypertension can be mitigated by strengthening the body’s antioxidant defences [4]. One of the most important microelements determining normal redox status in the body is Cu. Apart from its positive effects on the circulatory system through the regulation of blood clotting [10,11] and enhancement of immunity [12], Cu also acts as a cofactor, ensuring the activity of certain antioxidant enzymes, such as superoxide dismutase and ceruloplasmin [11,13,14]. Therefore, it seems likely that an adequate level of Cu in the body can limit hypertension-induced oxidative damage to cells and entire organs.

The bodies of humans and animals cannot accumulate copper reserves, so a steady source of it must be provided in the diet. The standard form of Cu in diet supplements for both people and animals is the inorganic form, CuCO_3_. This compound is relatively poorly absorbed by the body. Only 15% of Cu from inorganic forms is absorbed by the body, while the remaining 85% is excreted. For this reason, alternative forms of copper are being sought that would be better utilized and thus positively influence Cu-dependent metabolic processes, including antioxidant defences. One of the most interesting options seems to be Cu nanoparticles (CuNPs) [15]. The results of our previous study on rats showed that, owing to their specific physicochemical properties, CuNPs are better retained in the body than their standard counterparts [15]. Our research also demonstrated that replacing CuCO_3_ with CuNPs in the diet of rats reduced the oxidation and nitration of proteins and DNA in the blood [16], as well as lipid oxidation in the liver, while enhancing the antioxidant defences of the brain [17]. Therefore, it seems likely that CuNPs may have a stronger stimulatory effect on the antioxidant response than their macro counterparts, and thus can more effectively mitigate or even prevent negative changes induced by hypertension in the internal organs. However, there are no studies definitively confirming or refuting this supposition, and there are reports indicating that copper nanoparticles can be toxic for living organisms [18,19,20,21]. Copper, in the form of both inorganic compounds and nanoparticles, undergoes Fenton reactions, which result in the synthesis of free radicals. Therefore, an excessive Cu level in the body, especially in the form of more absorbable nanoparticles, can change its effect from antioxidative to pro-oxidative [11]. Thus, it can cause the oxidation of important cellular macromolecules, resulting in damage to the intestinal mucosa, lipid profile disturbances, and kidney dysfunction [22], which consequently exacerbates the changes caused by hypertension rather than eliminating them.

Therefore, we postulated that in hypertensive rats, there is an increased induction of oxidative processes that negatively affect the functionality of the liver, kidneys, intestine, and heart, and the partial or complete replacement of the standard form of Cu (CuCO_3_) in their diet with Cu nanoparticles (CuNPs) may reduce oxidation reactions and their adverse effects on these organs. The aim of the experiment was to test the effect of the partial (50%) or complete (100%) replacement of traditional CuCO_3_ in the diet of rats with copper in the form of CuNPs on selected blood biochemical parameters, redox status, and the histomorphometry of the liver, kidneys, intestine, and heart.

## 2. Materials and Methods

### 2.1. Drug and Chemicals

Metal-based copper nanoparticles (99.9% purity powder, 40–60 nm size, 12 m^2^/g specific surface area (SSA), spherical morphology, 0.19 g/cm^3^ bulk density, 8.9 g/cm^3^ true density) were purchased from SkySpring Nanomaterials Inc., (Houston, TX, USA). A stock solution (5 g/L) was prepared in rapeseed oil, and about 9% of the NPs were dissolved as Cu (II) ions; thus, the final suspension contained both NPs and released copper species. The zeta potential of the copper NP suspension was determined to be −30.3 mV (in phosphate buffered saline (PBS)) and −38.3 mV (pH 5), and the size was 104 nm (in rapeseed oil), determined by dynamic light scattering with a Zetasizer Nano ZS (Malvern Instruments, Malvern, UK) [16].

### 2.2. Experimental Protocol

Twenty-four-week-old normotensive Wistar–Kyoto (WKY/NCrl) rats from Charles River (Sulzfeld, Germany) were allocated randomly to 3 groups (*n* = 10 each) and individually fed experimental diets for 8 weeks under standard laboratory conditions. Three analogous groups of spontaneously hypertensive rats (SHR/NCrl) were formed (Charles River, Sulzfeld, Germany). Only male rats were studied to enable comparison with the previous experiments. The experiment was carried out in compliance with the European Guidelines for the Care and Use of Laboratory Animals and was approved by the Local Ethics Committee for Animal Experiments in Olsztyn Local Animal Care and Use Committee (Approval No. 90/2019; Olsztyn, Poland). The study was carried out in compliance with the ARRIVE guidelines. Every effort was made to minimize the suffering of the animals used in the experiment. The rats were fed additional copper in a standard daily dose of 6.5 mg/kg, either as carbonate salt (CuCO_3 6.5_) or metal NPs (NP_6.5_). The third group (CuCO_3 3.25_ + NP_3.25_) additionally received 3.25 mg of copper NPs plus 3.25 mg of copper carbonate. CuCO_3_ was added as a dietary ingredient in the mineral mixture, while the copper nanoparticles were supplied together with dietary rapeseed oil (for the safety of the person preparing the diets) [23]. Semi-purified diets were prepared as recommended by the American Institute of Nutrition [24].

### 2.3. Experimental Procedure

All physiological measurements were made separately for each animal (*n* = 8 for each group). At the end of the experiment, the rats were fasted for 24 h and anaesthetized intraperitoneally with ketamine and xylazine (K, 100 mg/kg body weight (BW); X, 10 mg/kg BW) according to the recommendations for the anaesthesia and euthanasia of experimental animals. Then, blood was collected from the vena cava and centrifuged at 3000× *g* for 10 min to separate the plasma, which was further stored at −80 °C until analysis. Finally, the rats were euthanized by cervical dislocation, and the heart, small intestine, liver, and kidney were removed. Samples of the heart, small intestine, liver, and kidney were preserved for histopathological examination, and homogenates from these organs were prepared and stored at −80 °C until analysis.

### 2.4. Biochemical Analyses of Plasma

The activity of alanine aminotransferase (ALT), aspartate aminotransferase (AST), creatine kinase (CK), and gamma-glutamyl transferase (GGT), as well as the content of creatinine (CREAT) and urea acid (UA), in the plasma were measured using an automatic biochemical analyser (Plasma Diagnostic Instruments Horiba, Kyoto, Japan).

### 2.5. Analysis of Redox Status

Indicators of antioxidant status, i.e., the activity of superoxide dismutase (SOD) and catalase (CAT) and the concentrations of malondialdehyde (MDA) and total glutathione (GSH + GSSG), were determined in the homogenates of the heart, small intestine, liver, and kidney according to the procedure described in [25].

### 2.6. Histological Examination of Organs

Samples of the heart, small intestine, liver, and kidney were cut in half lengthwise and fixed for 24 h in 5% formalin, pH = 7.2. Within 24 h, the fixed tissue fragments were passed through increasing concentrations of alcohol solutions, acetone, and xylene into paraffin blocks in a tissue processor (Leica TP-20, Leica Biosystems, Deer Park, TX, USA). Paraffin-embedded microscope sections 5 μm thick were stained with haematoxylin and eosin (HE staining). A morphometric evaluation of the organs was carried out using a computer-assisted microscopic image analysis system. The system included a light microscope (Nikon Eclipse E600, Nikon Europe BV, Amsterdam, The Netherlands) with a digital camera (Nikon DS-Fi1, Nikon Europe BV, Amsterdam, The Netherlands) and a PC with image-analysis software (NIS-Elements BR-2.20, Laboratory Imaging, Nikon Europe BV, Amsterdam, The Netherlands).

### 2.7. Data Analysis and Statistics

For the analysis of parameters, individual rats (*n* = 10) were considered as experimental units. The data were subjected to two-way ANOVA to examine the following effects: (a) C, the main effect of the Cu source (CuCO_3 6.5_ or CuNP_6.5_ or CuCO_3 3.25_ +CuNP_3.25_); (b) R, the main effect of the rat strain (normotensive WKY or spontaneously hypertensive SHR); and (c) the interaction between C and R (C×R). When a significant C×R interaction effect was noted (F test), the treatment means were separated using the post-hoc Tukey test. All calculations were performed using the generalized linear model (GLM) procedures of the STATISTICA software ver. 13.1 (StatSoft Inc., 2016, Tulsa, OK, USA). Data variability was expressed as means with a pooled standard error of the mean (SEM), and *p* < 0.05 was considered statistically significant.

## 3. Results

### 3.1. Effect of Blood Pressure (R)

The activity of ALT and CK was higher in the plasma of the SHR rats in comparison to the WKY rats (*p* = 0.035 and *p* = 0.027, respectively), while the CREAT level was lower (*p* = 0.014; Table 1). In comparison to the WKY rats, the SHR rats had higher SOD activity in the heart and liver (*p* = 0.024 and *p* < 0.001, respectively; Table 2 and Table 3) and a lower activity of this enzyme in the kidney (*p* = 0.012; Table 4). In addition, CAT activity in the liver and kidney was higher in the SHR rats (*p* < 0.001 and *p* = 0.047, respectively; Table 3 and Table 4). The GSH + GSSG level in the kidney of SHR rats was lower (*p* = 0.031) than that of WKY rats (Table 4).

### 3.2. Effect of Cu Source (C)

Irrespective of blood pressure, 50% or 100% replacement of the standard form of Cu (CuCO_3_) with CuNPs in the diet of rats increased the activity of AST and CK in the plasma (*p* = 0.002 and *p* = 0.003, respectively). In the case of 100% replacement of CuCO_3_ with CuNPs, a reduction in ALT activity (*p* = 0.023) was noted in the plasma of rats relative to those for which the CuCO_3_ additive was only partially (50%) replaced with CuNPs (Table 1).

The administration of a diet in which the standard addition of CuCO_3_ was partially (50%) or completely replaced with CuNPs resulted in an increase in SOD activity (*p* < 0.001) in the heart. In addition, 50% replacement of CuCO_3_ with CuNPs was found to reduce CAT activity in the heart tissue, while 100% replacement increased it (*p* = 0.007) (Table 2).

The liver of rats receiving a diet in which CuCO_3_ was fully replaced with CuNPs showed higher levels of MDA and GSH + GSSG (*p* = 0.047 and *p* = 0.002, respectively). SOD activity (*p* < 0.001) was also increased in the liver of rats receiving a diet in which CuCO_3_ was 50% replaced with CuNPs; however, it was reduced when CuCO_3_ was 100% replaced with CuNPs. Both 50% and 100% replacement of CuCO_3_ with CuNPs reduced CAT activity (*p* < 0.001) in the liver of rats (Table 3).

The replacement of 50% or 100% of the CuCO_3_ additive in the diet of rats with CuNPs also resulted in an increase in the GSH + GSSG level and SOD activity (*p* < 0.001 for both) in the kidney. In the case of GSH + GSSG, this effect was more pronounced when CuCO_3_ was completely replaced with CuNPs, while the effect on SOD activity was more pronounced in the case of 50% replacement. Only 100% replacement of the standard form of copper (CuCO_3_) with CuNPs caused an increase in CAT activity (*p* = 0.008) in the kidney (Table 4). In addition, there was a reduction in the GSH + GSSG level in the small intestinal tissue of rats receiving a diet in which CuCO_3_ was completely replaced with CuNPs, (*p* < 0.001; Table 5).

### 3.3. Histology of Organs

**Figure 1:**
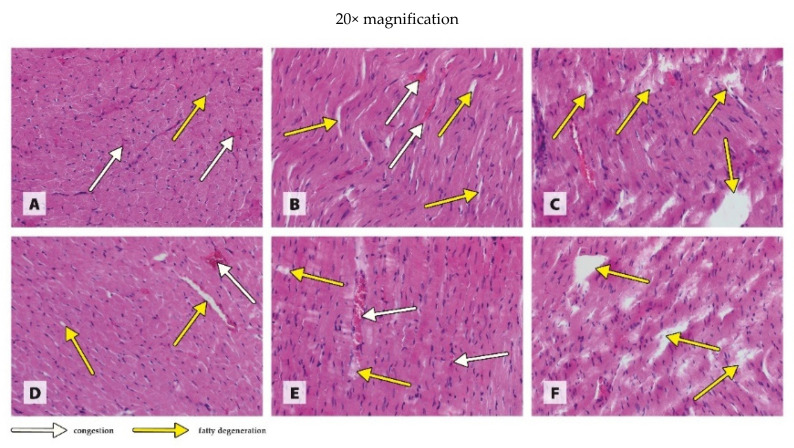

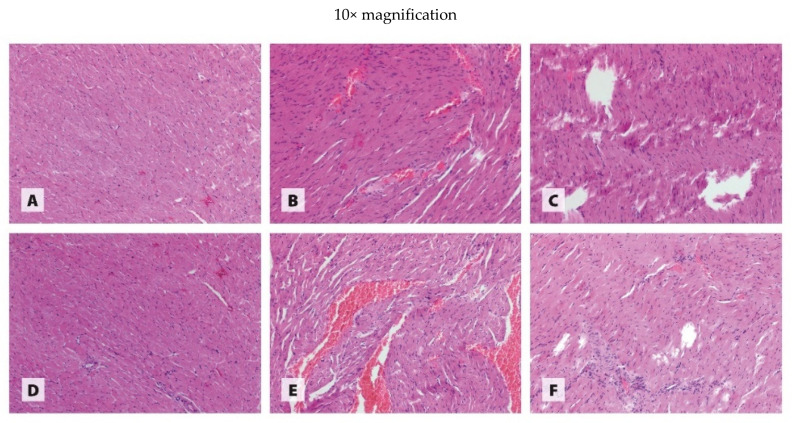
Morphological effects of different blood pressure and Cu sources on the heart muscle of rats. Treatments: (**A**) WKY CuCO_3 6.5_, (**B**) WKY CuCO_3 3.25_ + CuNP_3.25_, (**C**) WKY CuNP_6.5_, (**D**) SHR CuCO_3 6.5_, (**E**) SHR CuCO_3 3.25_ + CuNP_3.25_, and (**F**) SHR CuNP_6.5_.

**Figure 2:**
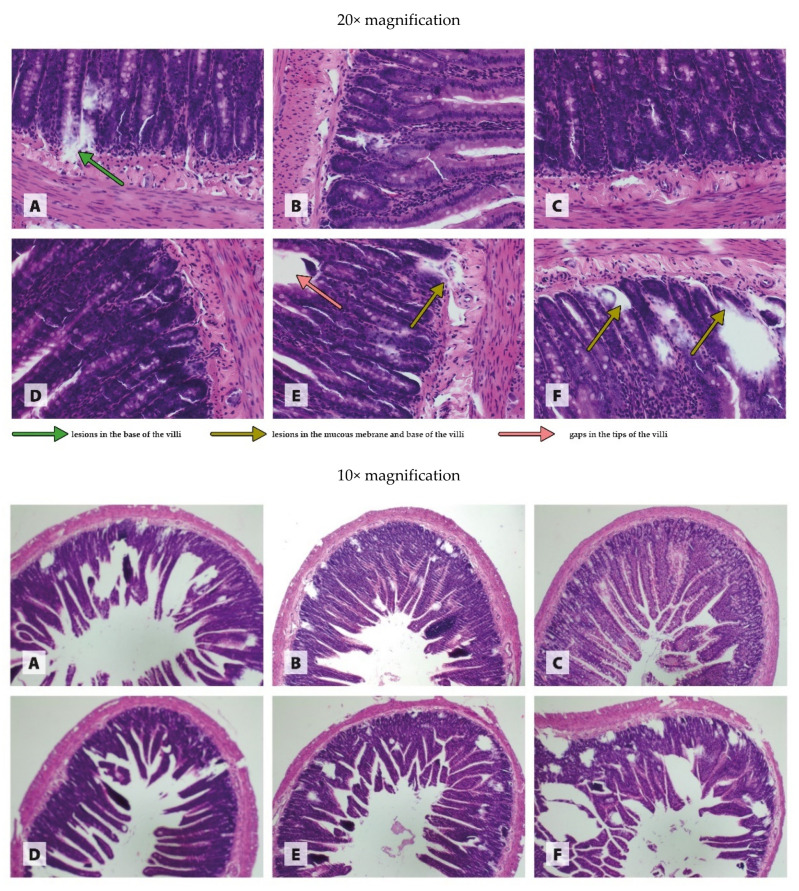
Morphological effects of different blood pressure and Cu sources on the small intestine of rats. Treatments: (**A**) WKY CuCO_3 6.5_, (**B**) WKY CuCO_3 3.25_ + CuNP_3.25_, (**C**) WKY CuNP_6.5_, (**D**) SHR CuCO_3 6.5_, (**E**) SHR CuCO_3 3.25_ + CuNP_3.25_, and (**F**) SHR CuNP_6.5_.

**Figure 3:**
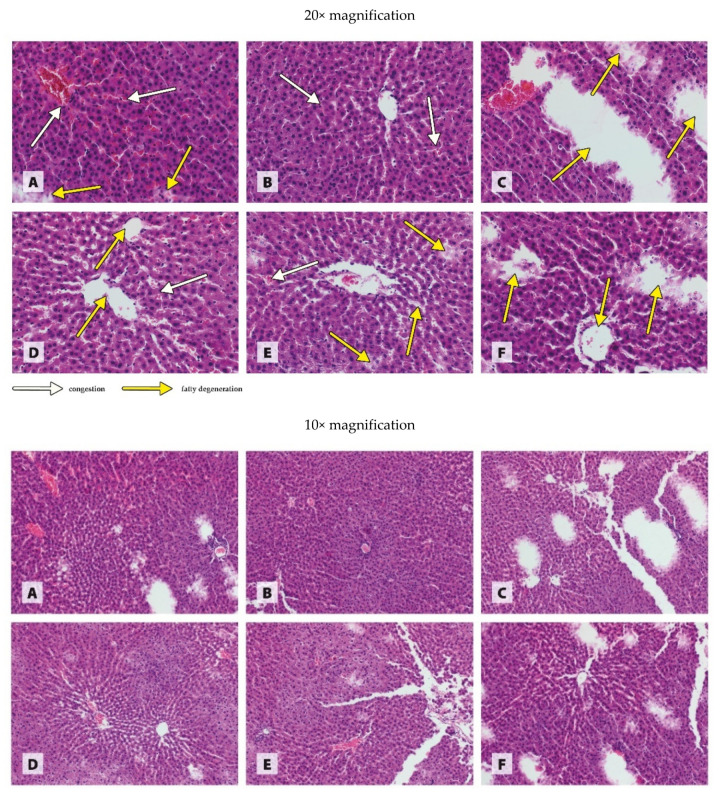
Morphological effects of different blood pressure and Cu sources on the liver of rats. Treatments: (**A**) WKY CuCO_3 6.5_, (**B**) WKY CuCO_3 3.25_ + CuNP_3.25_, (**C**) WKY CuNP_6.5_, (**D**) SHR CuCO_3 6.5_, (**E**) SHR CuCO_3 3.25_ + CuNP_3.25_, and (**F**) SHR CuNP_6.5_.

**Figure 4:**
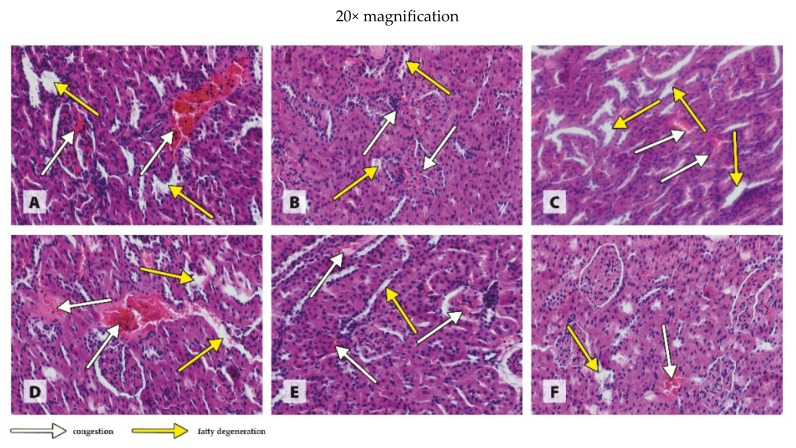

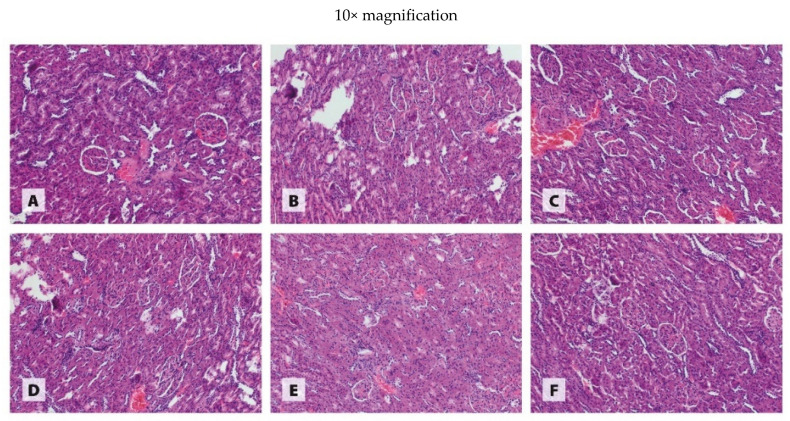
Morphological effects of different blood pressure and Cu sources on the kidney of rats. Treatments: (**A**) WKY CuCO_3 6.5_, (**B**) WKY CuCO_3 3.25_ + CuNP_3.25_, (**C**) WKY CuNP_6.5_, (**D**) SHR CuCO_3 6.5_, (**E**) SHR CuCO_3 3.25_ + CuNP_3.25_, and (**F**) SHR CuNP_6.5_.

The histological images in Figure 1, Figure 2, Figure 3 and Figure 4 are presented at two magnifications; the intended purpose of this was to accurately represent the histological changes (20× magnification) and to visualize the extent of these changes against unchanged tissue (10× magnification).

The histological image of the heart muscle from WKY rats receiving 6.5 mg CuCO_3_ in their diet revealed low-grade generalized congestion and fatty degeneration (Figure 1A). The generalized congestion of the tissue was similarly noted in the WKY rats receiving a diet in which the traditional form of CuCO_3_ was 50% replaced with CuNPs; however, local foci of fatty degeneration were much more numerous (Figure 1B). In the heart muscle of WKY rats receiving a diet in which the traditional Cu additive was completely replaced with CuNPs, there were only numerous extensive foci of fatty degeneration (Figure 1C). In the SHR rats receiving 6.5 mg CuCO_3_ in their diet, as in the case of the WKY rats, the histological image of the heart muscle revealed low-grade congestion and generalized fatty degeneration (Figure 1D). The replacement of 50% of the traditional CuCO_3_ with CuNPs resulted in severe local congestion and numerous small foci of fatty degeneration in the heart muscle of SHR rats (Figure 1E). On the other hand, 100% replacement with CuNPs resulted only in numerous foci of fatty degeneration in the tissue (Figure 1F).

The histopathological examination of the intestine revealed generally isolated extensive lesions in the base of the villi in WKY rats receiving the recommended copper additive in the standard form of CuCO_3_ (Figure 2A). The histological image of the intestine of WKY rats fed a diet in which 50% of the traditional CuCO_3_ was replaced with CuNPs showed no significant pathological changes in the tissue (Figure 2B), as in the case of the WKY rats in which CuCO_3_ was fully replaced with CuNPs (Figure 2C). The histological image of the intestine of SHR rats receiving the recommended Cu additive in the standard form of CuCO_3_ showed no pathological changes (Figure 2D). The partial (50%) replacement of the traditional CuCO_3_ additive with CuNPs in the diet of SHR rats revealed isolated lesions in the mucous membrane and the base of the villi, as well as single gaps in the tips of the villi (Figure 2E). A diet in which the standard form of CuCO_3_ was fully replaced with CuNPs resulted in numerous lesions in the mucous membrane and the base of the villi (Figure 2F).

The histopathological examination of the liver of rats revealed generalized congestion in the tissue and isolated foci of fatty degeneration in the WKY group receiving the recommended copper additive in the standard form of CuCO_3_ (Figure 3A). The histological image of the liver of WKY rats receiving a diet in which 50% of the standard form of copper was replaced with CuNPs indicated the generalized congestion of the tissue (Figure 3B). The liver of WKY rats receiving a diet in which CuCO_3_ was completely replaced with CuNPs showed numerous large foci of fatty degeneration (Figure 3C). The liver of SHR rats receiving a copper additive in the standard form of CuCO_3_ exhibited characteristics of fatty degeneration in combination with isolated foci of congestion (Figure 3D). The partial (50%) replacement of CuCO_3_ with CuNPs in the diet of SHR rats resulted in the appearance of scattered small foci of fatty degeneration in combination with isolated large foci, as well as low-grade congestion in the tissue (Figure 3E). The complete replacement of CuCO_3_ with CuNPs caused numerous extensive foci of fatty degeneration in the liver (Figure 3F).

The histopathological evaluation of the kidney revealed generalized congestion of the tissue in combination with local foci of fatty degeneration, both in the WKY rats receiving a diet that contained the recommended copper additive in the standard form of CuCO_3_ or a diet in which 50% or 100 % of the CuCO_3_ was replaced with CuNPs (Figure 4A–C).The histological image of the kidney of SHR rats receiving the recommended CuCO_3_ additive showed numerous local foci of congestion in combination with local foci of fatty degeneration (Figure 4D). Both 50% and 100% replacement of CuCO_3_ with CuNPs in the diet of SHR rats caused local foci of fatty degeneration in combination with generalized congestion of the kidney (Figure 4E,F), which was less severe in the case of the higher dose (Figure 4F).

It seems important to emphasize that the pharmacological preparations used to euthanize the rats may have had some effect on the results of the biochemical analysis of the blood and the occurrence and severity of pathological changes in the organs. While this hypothesis cannot be verified at this stage of research, a thorough analysis of the results requires that this potential influence be mentioned.

## 4. Discussion

The available literature indicates that hypertension can lead to damage to numerous internal organs. The most susceptible, however, seem to be the heart, central and peripheral arteries, kidneys, central nervous system, and eyes [26,27]. There are also reports suggesting that elevated blood pressure can adversely affect the liver and even the intestines [3]. The chronic increase in blood pressure observed in hypertension triggers a number of initially compensatory pathophysiological and neurohormonal changes, which ultimately lead to damage to the heart and subsequently other target organs [26]. The results of the biochemical analyses in our study confirmed the negative effect of hypertension on the heart and liver, as indicated by the increased activity of the enzymes CK and ALT in the plasma. CK exhibits high activity in heart cells because it plays a key role in cellular energy metabolism via the rapid regeneration of ATP from phosphocreatine in the vicinity of subcellular localizations with a high energy demand [1]. ALT is synthesized mainly in the liver [28]. Both CK and ALT are intracellular enzymes; in physiological conditions, they exhibit a very low level of activity in the blood plasma. Organ damage results in an increased release of these enzymes into the bloodstream, where their activity is significantly amplified [1,28]. The histopathological examination in our study confirmed an increase in pathological changes in the liver due to hypertension; however, surprisingly, it did not demonstrate that this experimental factor had a negative effect on the histological image of the heart. Moreover, the histopathological examination revealed less severe pathological changes in the intestine and kidney of hypertensive rats in comparison to normotensive individuals. The absence of a negative effect of elevated blood pressure on the kidneys was additionally confirmed by the reduced plasma CREAT level relative to the rats with normal blood pressure. This is in conflict with the available literature, which indicates that chronic arterial hypertension leads to cell damage, atherosclerosis, nephron loss, and a further increase in systemic hypertension [26]. The lack of changes in the histopathological image may be due to the young age of the rats. It is possible that over time, the effects of hypertension on the histological image might have changed. However, it seems likely that the body adapts to some extent to unfavourable conditions, such as chronic hypertension, which does not result in pronounced changes in organ histology. Only when a certain critical point has been reached do unfavourable changes take place in the morphology and function of the kidneys.

The results of our study in rats showed that both 50% and 100% replacement of the standard Cu source in the diet with CuNPs resulted in damage to the heart muscle, as indicated by increased CK activity in the tissue. Similarly, Jing et al. [29] noted a linear increase in the CK-MB level in rats receiving CuONPs intraperitoneally (15–25 nm) in amounts of 0.5, 5, and 50 mg/kg BW. However, they found no changes of concern in the histopathological image of the heart as an effect of CuONPs, even at the highest dose (50 mg/kg BW). This is in contrast to our results, which indicate that both 50% and 100% replacement of CuCO_3_ with CuNPs increase fatty degeneration, and 50% replacement of CuCO_3_ with CuNPs additionally increases congestion in the hearts of both normotensive and hypertensive rats. The discrepancies observed between the results of our own research and that of Jing et al. [29] may be due to a variety of factors, such as the form of NPs used (CuNPs vs. CuONPs), their size (40 nm vs. 15–25 nm), the dosage of nanoparticles (3.25 or 6.5 mg/kg diet vs. 0.5, 5 and 50 mg/kg BW), or the route of administration (in the diet vs. intraperitoneal). However, it seems that the most important factor is the period during which the nanoparticles were used. Jing et al. [29] used a single injection, while in our study, the rats received CuNPs regularly for a long period. A single administration of CuNPs to rats, even at a high dose (50 mg/kg), may have been insufficient to cause visible histopathological changes similar to those resulting from a long period of administration of the experimental factor. It should be noted, however, that both our own study and that of Jing et al. [29] indicate that CuNPs can exhibit cardiotoxicity. Consequently, the inclusion of CuNPs in the diet of hypertensive rats may exacerbate, rather than mitigate, heart damage.

According to the available literature, Cu in the form of nanoparticles can also have toxic effects on the liver [30]. Eid et al. [30] demonstrated the increased activity of AST and ALT in rats receiving CuONPs (50 nm) orally through a tube in the amount of 5 mg/kg BW for 30 days. Similarly, Mohammadyari et al. [31], after the intraperitoneal administration of 100, 200, or 400 ppm CuONPs (50 nm) to rats for 15 days, observed a linear increase in AST and ALT activity relative to the controls, although the effect was statistically significant only in rats receiving the highest dose of CuONPs. The results of our study confirm earlier reports of a possible hepatotoxic effect of CuNPs, as AST activity was shown to increase as a result of both levels of CuNPs in the diet of rats. In addition, 100% replacement of CuCO_3_ with CuNPs in the diet increased ALT activity in the plasma, which indicates that as the level of CuNPs in the diet is increased, the severity of tissue damage increases. This is fully confirmed by the histological image of the liver from the experimental rats. It should be noted, however, that in hypertensive rats, both 50% and 100% replacement of CuCO_3_ with CuNPs caused visible morphological changes in the liver, and their severity increased with the dose of nanoparticles. In normotensive rats, pathological changes only appeared when CuCO_3_ was entirely replaced with CuNPs. The results of the histopathological examination of the small intestine were also interesting; they showed that 50% and 100% replacement of CuCO_3_ with CuNPs in the diet of normotensive rats did not worsen, and even improved, the morphology of the intestines, whereas in hypertensive rats, it increased the severity of the pathological changes in the form of damage to the basement membrane and villi. In light of these findings, it seems likely that the body’s response to CuNPs may depend on the blood pressure level. Hypertension seems to make the liver and intestines more sensitive to the toxic effects of CuNPs, most likely due to an overall increase in metabolism, and thus in the metabolism of CuNPs.

The results of studies by Mohammadyari et al. [31] and Liao and Liu [32] indicate that Cu in the form of nanoparticles may exhibit nephrotoxic properties. Mohammadyari et al. [31], following the intraperitoneal administration of CuNPs (50 nm) to rats in amounts of 100, 200, and 400 ppm for 15 days, noted a significant increase in the plasma CREAT level only at 400 ppm. Liao and Liu [32], who administered CuNPs (25 nm) orally to rats through a tube at 100 and 200 mg/kg BW, noted an increase in the CREAT level, disturbances in the expression of many kidney profile genes, and the extensive necrosis of the proximal tubules of the kidneys in the case of the higher dose. Thus, the results of both of these experiments [31,32] suggest that Cu in the form of nanoparticles only has an adverse effect on the kidneys when used in relatively high doses. This is confirmed by the results of our study, in which rats received the recommended level of Cu or half that level in the form of CuNPs, as indicated by the lack of a negative effect on the plasma level of CREAT in both experimental treatments. Moreover, the histological examination of the kidney showed that both 50% and 100% replacement of CuCO_3_ with CuNPs in the diet had no effect on this organ in both normotensive and hypertensive rats.

Internal organ damage observed in patients with hypertension is believed to be an effect of haemodynamic overload caused by increased blood pressure [1]. However, an increasingly common hypothesis states that hypertension, by accelerating overall metabolism, increases the synthesis of free radicals [4,6]. These in turn weaken the body’s antioxidant defences and lead to the oxidation of important cellular structures, such as lipids, proteins, and nucleic acids, and thus to organ damage [4,6]. Antioxidant enzymes, including SOD and CAT, play an important role in the body’s defences against the harmful effects of free radicals. These enzymes have complementary effects. SOD catalyses the conversion of superoxide radical to hydrogen peroxide, which is then broken down by catalase to chemically neutral water and oxygen molecules [33]. The literature indicates that chronic arterial hypertension can cause the impairment of the body’s antioxidant defences, manifested as a reduced activity of antioxidant enzymes accompanied by an increased synthesis of hydrogen peroxide and lipid hydroperoxides [9]. The results of our study in rats showed that elevated blood pressure increased SOD activity in the heart and liver and CAT activity in the liver and kidney, which were accompanied by a reduction in SOD activity in the kidney. These findings suggest that hypertension beneficially stimulated the antioxidant status of the heart, liver, and kidney of rats, which sufficiently protected them against lipid peroxidation, as evidenced by the lack of an increase in the MDA level in these tissues. Therefore, it can be assumed that the body is to some extent able to adapt to negative conditions by improving its defence mechanisms, and the unfavourable biochemical changes observed in the heart and liver and the pathologies in the histology of the liver of the hypertensive rats were not due to increased oxidative stress, but to haemodynamic overload induced by the elevated pressure of blood flowing through the organs.

Copper, as a cofactor of Cu/Zn SOD and by binding to ceruloplasmin, enhances the body’s antioxidant defences. On the other hand, as a transition metal, it undergoes Fenton and Haber–Weiss reactions, thereby increasing the synthesis of free radicals. According to the available literature, CuNPs, owing to their better bioavailability and greater reactivity, can have a much stronger effect on redox status than their standard counterparts, such as CuCO_3_ [16,17]. Our study showed that both 50% and 100% replacement of CuCO_3_ with CuNPs in the diet of rats increased SOD activity in the heart. The complete replacement of CuCO_3_ with CuNPs additionally increased CAT activity in this tissue. This suggests that CuNPs beneficially stimulate antioxidant defences, which is additionally evidenced by the lack of negative changes in the glutathione and MDA levels. Similarly, Jing et al. [29] found no deterioration of antioxidant defences in the heart muscle following a single application of CuONPs to healthy rats in the range of 0.5–50 mg/kg BW. Our results also showed no disturbance of the redox status in the intestine of normotensive and hypertensive rats resulting from the inclusion of CuNPs in the diet. While a reduction in the glutathione level was noted in the case of 100% replacement of CuCO_3_ with CuNPs, due to the lack of a negative effect on the activity of antioxidant enzymes or an increase in the lipid peroxidation processes, this could not be linked to the disturbances in the antioxidant response. This suggests that the heart and intestinal damage observed in our study in the groups receiving CuNPs was not induced by free radicals.

According to the available literature, the liver is particularly susceptible to the toxic effects of CuNPs [30,34]. Researchers link the mechanism of toxicity of CuNPs to increased pro-oxidative processes taking place in the body [30,34,35]. These can lead to the oxidation of cellular macromolecules and thus to organ damage through a loss of the physiological activity of proteins, the accumulation of mutations in genetic material, and a loss of fluidity in biological membranes [17]. Eid et al. [30] reported that the oral administration of CuONPs to rats at 5 mg/kg BW for 30 days adversely affected the antioxidant defences of the liver and increased lipid peroxidation, as evidenced by reduced SOD and CAT activity, a reduced GSH level, and an increase in the level of MDA. Similar results were obtained by Anreddy [34] following the administration of CuONPs (<50 nm) to rats orally through a tube in amounts of 5 and 50 mg/kg BW/day; the degree of the disturbance in redox status in the liver increased with the dose. Our results are in agreement with the reports of the authors cited [30,34], as they showed that 50% replacement of CuCO_3_ with CuNPs reduced CAT activity while increasing SOD activity in the liver. The uncoordinated activity of the antioxidant enzymes SOD and CAT observed in the liver of rats receiving the lower level of CuNPs may thus increase oxidative stress, as the increased dismutation of O_2_^−^ by SOD significantly increases the concentration of H_2_O_2_, which can lead to tissue damage if there is no compensation by the reduced CAT activity [36]. Moreover, in the case of 100% replacement of CuCO_3_ with CuNPs, there was a reduction in CAT activity accompanied by an increase in MDA and GSH levels in the tissue. While the increase in the level of glutathione, an endogenous antioxidant, is somewhat surprising and difficult to explain, the increase in lipid peroxidation and the accompanying reduction in CAT activity indicates a disturbance in the antioxidant defences of the liver. This suggests that the liver damage observed in our study, confirmed by the biochemical analysis and histopathological examination, may be directly linked to the pro-oxidative effects of CuNPs. Moreover, it is highly likely that the CuNPs used in hypertensive individuals, instead of mitigating the adverse changes in the morphology and function of the liver caused by hypertension, may only exacerbate them. Nanoparticles are very small in size; owing to this, they have the ability to penetrate biological membranes, block ion channels, inhibit enzymatic proteins, and interact with genetic material, which may result in the development of oxidative stress [37]. Research by Liu et al. [38], which used three different sizes of silver nanoparticles (AgNPs; 5, 20, and 50 nm), showed that smaller nanoparticles penetrate cells more easily than larger ones, which may result in stronger biological and toxic effects. In addition, Lee et al. [20] reported the increased biodistribution of copper nanoparticles (CuNPs—25 nm) relative to copper microparticles (CuMPs, 14–25 μm) in rats. Moreover, Wen et al. [39] found that larger-sized particles can be removed from the organism more easily than smaller ones. Based on the above reports, it can be assumed that the nanoparticles with an average size of 40 nm used in our experiment were not evenly distributed in all tissues; thus, their pro-oxidative effect on the organs to which they were not distributed or were delivered in a very small amount was reduced. In addition, the fact that a CuNPs-dependent deterioration in redox status was observed only in the liver seems to support this assumption. The liver, as the organ responsible for the biotransformation processes of xenobiotics, was exposed to the greatest impact of the nanoparticles used among all the organs that were the subject of our research.

Lei et al. [35] showed that in addition to the liver, the kidneys are also susceptible to the negative effects of CuNPs. In the liver of rats receiving CuONPs (25 nm) orally through a tube for five consecutive days in amounts of 100 or 200 mg/kg BW, the MDA level was increased in the case of the higher dose [35]. Our previous research also found that the replacement of the recommended level of CuCO_3_ with CuNPs in the diet of rats adversely affected antioxidant defences and increased lipid peroxidation in the kidney [17]. The results of the present study, however, do not confirm these findings, as SOD and GSH activity were increased in the kidney of rats in the case of both 50% and 100% replacement of CuCO_3_ with CuNPs, irrespective of blood pressure. The complete replacement of CuCO_3_ with CuNPs additionally increased CAT activity in the kidney. This suggests that the experimental factor had a beneficial effect on antioxidant status, which increased with the level of CuNPs. The simultaneous improvement in the biochemical parameters of the kidneys due to the inclusion of CuNPs in the diet suggests that they can be used to mitigate the negative effects of hypertension in this tissue.

## 5. Conclusions

The results of our study indicate that the replacement of CuCO_3_ with CuNPs in the diet of rats may exacerbate the negative changes induced by hypertension in the heart, liver, and intestines. Nevertheless, it seems that it is only in the case of the liver where the changes observed may be due to an increase in oxidative reactions resulting from the inclusion of CuNPs in the diet of rats.

## Figures and Tables

**Table 1:** Blood biochemical parameters.

Dietary Treatment ^1^		AST U/L	ALT U/L	GGT U/L	CREAT umol/L	UA umol/L	CK U/L
WKY CuCO_3 6.5_		159.85	4.14	0.93	76.54	44.20 ^c^	1124.2
SHR CuCO_3 6.5_		138.27	6.27	0.80	72.95	77.68 ^b^	1173.4
WKY CuCO_3 3.25_ + CuNP_3.25_		207.58	5.61	0.89	77.74	70.31 ^b^	1019.8
SHR CuCO_3 3.25_ + CuNP_3.25_		185.84	7.54	0.92	64.58	31.47 ^c^	1792.5
WKY CuNP_6.5_		195.62	4.99	0.61	96.87	118.53 ^a^	1551.4
SHR CuNP_6.5_		175.21	4.64	0.94	41.86	69.64 ^b^	1754.2
SEM		0.645	0.024	0.089	0.866	0.985	0.766
Blood pressure (R)							
	WKY	187.69	4.91 ^b^	0.81	83.72 ^a^	77.68	1231.8 ^b^
	SHR	166.44	6.15 ^a^	0.89	59.80 ^b^	59.60	1573.4 ^a^
Cu source (C)							
	CuCO_3 6.5_	149.06 ^b^	5.20 ^ab^	0.87	74.75	60.94	1148.8 ^c^
	CuCO_3 3.25_ + CuNP_3.25_	196.71 ^a^	6.57 ^a^	0.90	71.16	50.89	1406.2 ^b^
	CuNP_6.5_	185.41 ^a^	4.82 ^b^	0.78	69.36	94.08	1652.8 ^a^
*p* value							
R effect		0.062	0.035	0.876	0.014	0.006	0.027
C effect		0.002	0.023	0.072	0.681	<0.001	0.003
R × C interaction		0.077	0.052	0.135	0.357	0.048	0.244

^1^ WKY CuCO_3 6.5_—normotensive rats fed 6.5 mg/kg copper carbonate salt; SHR CuCO_3 6.5_—hypertensive rats fed 6.5 mg/kg copper carbonate salt; WKY CuCO_3 3.25_ + CuNP_3.25_—normotensive rats fed 3.25 mg/kg CuNPs plus 3.25 mg/kg copper carbonate salt; SHR CuCO_3 3.25_ + CuNP_3.25_—hypertensive rats fed 3.25 mg/kg CuNPs plus 3.25 mg/kg copper carbonate salt; WKY CuNP_6.5_—normotensive rats fed 6.5 mg/kg CuNPs; SHR CuNP_6.5_—hypertensive rats fed 6.5 mg/kg CuNPs. ^a, b, c^ Means within a column with different superscript letters are significantly different (*p* < 0.05); differences between groups (WKY CuCO36.5, SHR CuCO_3 6.5_, WKY CuCO_3 3.25_ + CuNP _3.25_, SHR CuCO_3 3.25_ + CuNP_3.25_, WKY CuNP_6.5_, SHR CuNP_6.5_) are indicated with superscripts ^a, b, c^ only in the case of a statistically significant R × C interaction (*p* < 0.05). SEM—pooled standard error of mean (standard deviation for all rats divided by the square root of the number of rats, *n* = 60); AST—aspartate aminotransferase; ALT—alanine aminotransferase; GGT—gamma-glutamyl transferase; CREAT—creatinine; UA—urea acid; CK—creatine kinase.

**Table 2:** Redox status in heart tissue.

Dietary Treatment ^1^		MDA µmol/g	GSH + GSG umol/kg	SOD U/g	CAT U/g
WKY CuCO_3 6.5_		0.984 ^ab^	13.237 ^c^	16.89	219.32
SHR CuCO_3 6.5_		0.787 ^c^	12.615 ^c^	26.30	234.69
WKY CuCO_3 3.25_ + CuNP_3.25_		0.942 ^b^	20.361 ^a^	37.01	173.76
SHR CuCO_3 3.25_ + CuNP_3.25_		0.976 ^ab^	17.411 ^b^	37.01	176.36
WKY CuNP_6.5_		1.209 ^a^	15.767 ^b^	38.53	227.07
SHR CuNP_6.5_		1.095 ^ab^	18.655 ^ab^	32.83	293.46
SEM		0.048	0.145	0.231	0.645
Blood pressure (R)					
	WKY	0.963	16.46	26.95 ^a^	196.54
	SHR	0.881	16.23	31.65 ^b^	205.52
Cu source (C)					
	CuCO_3 6.5_	0.885	12.926	21.59 ^b^	227.01 ^b^
	CuCO_3 3.25_ + CuNP_3.25_	0.959	18.886	37.01 ^a^	175.06 ^c^
	CuNP_6.5_	1.152	17.211	35.68 ^a^	260.27 ^a^
*p* value					
R effect		0.042	0.752	0.024	0.974
C effect		0.006	0.014	<0.001	0.007
R × C interaction		0.033	0.047	0.054	0.258

^1^ WKY CuCO_3 6.5_—normotensive rats fed 6.5 mg/kg copper carbonate salt; SHR CuCO_3 6.5_—hypertensive rats fed 6.5 mg/kg copper carbonate salt; WKY CuCO_3 3.25_ + CuNP_3.25_—normotensive rats fed 3.25 mg/kg CuNPs plus 3.25 mg/kg copper carbonate salt; SHR CuCO_3 3.25_ + CuNP_3.25_—hypertensive rats fed 3.25 mg/kg CuNPs plus 3.25 mg/kg copper carbonate salt; WKY CuNP_6.5_—normotensive rats fed 6.5 mg/kg CuNPs; SHR CuNP_6.5_—hypertensive rats fed 6.5 mg/kg CuNPs. ^a,b,c^ Means within a column with different superscript letters are significantly different (*p* < 0.05); differences between groups (WKY CuCO_3 6.5_, SHR CuCO_3 6.5_, WKY CuCO_3 3.25_ + CuNP _3.25_, SHR CuCO_3 3.25_ + CuNP_3.25_, WKY CuNP_6.5_, SHR CuNP_6.5_) are indicated with superscripts only in the case of a statistically significant R × C interaction (*p* < 0.05). SEM—pooled standard error of mean (standard deviation for all rats divided by the square root of the number of rats, *n* = 60); MDA—malondialdehyde; GSH + GSSG—total glutathione; SOD—superoxide dismutase; CAT—catalase.

**Table 3:** Redox status in liver tissue.

Dietary Treatment ^1^		MDA µmol/g	GSH + GSG umol/kg	SOD U/g	CAT U/g
WKY CuCO_3 6.5_		2.014	18.427	20.058	909.17
SHR CuCO_3 6.5_		1.471	17.313	23.621	1453.96
WKY CuCO_3 3.25_ + CuNP_3.25_		1.605	20.706	25.272	342.32
SHR CuCO_3 3.25_ + CuNP_3.25_		1.504	15.903	47.646	549.53
WKY CuNP_6.5_		1.993	22.078	14.240	517.79
SHR CuNP_6.5_		2.253	25.816	16.657	535.09
SEM		0.036	0.108	0.078	0.364
Blood pressure (R)					
	WKY	1.809	19.566	22.665 ^b^	625.74 ^b^
	SHR	1.488	16.608	35.633 ^a^	1001.75 ^a^
Cu source (C)					
	CuCO_3 6.5_	1.742 ^b^	17.870 ^b^	21.839 ^b^	1181.56 ^a^
	CuCO_3 3.25_ + CuNP_3.25_	1.555 ^b^	18.305 ^b^	36.459 ^a^	445.92 ^b^
	CuNP_6.5_	2.123 ^a^	23.947 ^a^	15.448 ^c^	526.44 ^b^
*p* value					
R effect		0.058	0.259	<0.001	<0.001
C effect		0.047	0.002	<0.001	<0.001
R × C interaction		0.057	0.069	0.693	0.386

^1^ WKY CuCO_3 6.5_—normotensive rats fed 6.5 mg/kg copper carbonate salt; SHR CuCO_3 6.5_—hypertensive rats fed 6.5 mg/kg copper carbonate salt; WKY CuCO_3 3.25_ + CuNP_3.25_—normotensive rats fed 3.25 mg/kg CuNPs plus 3.25 mg/kg copper carbonate salt; SHR CuCO_3 3.25_ + CuNP_3.25_—hypertensive rats fed 3.25 mg/kg CuNPs plus 3.25 mg/kg copper carbonate salt; WKY CuNP_6.5_—normotensive rats fed 6.5 mg/kg CuNPs; SHR CuNP_6.5_—hypertensive rats fed 6.5 mg/kg CuNPs. ^a,b,c^ Means within a column with different superscript letters are significantly different (*p* < 0.05); differences between groups (WKY CuCO36.5, SHR CuCO_3 6.5_, WKY CuCO_3 3.25_ + CuNP _3.25_, SHR CuCO_3 3.25_ + CuNP_3.25_, WKY CuNP_6.5_, SHR CuNP_6.5_) are indicated with superscripts only in the case of a statistically significant R × C interaction (*p* < 0.05). SEM—pooled standard error of mean (standard deviation for all rats divided by the square root of the number of rats, *n* = 60); MDA—malondialdehyde; GSH + GSSG—total glutathione; SOD—superoxide dismutase; CAT—catalase.

**Table 4:** Redox status in kidney tissue.

Dietary Treatment ^1^		MDA µmol/g	GSH + GSG umol/kg	SOD U/g	CAT U/g
WKY CuCO_3 6.5_		1.665^c^	0.709	16.65	531.44
SHR CuCO_3 6.5_		2.625 ^b^	0.323	13.31	629.08
WKY CuCO_3 3.25_ + CuNP_3.25_		3.059 ^a^	2.677	49.49	583.92
SHR CuCO_3 3.25_ + CuNP_3.25_		2.081 ^b^	2.381	31.78	602.38
WKY CuNP_6.5_		2.252 ^b^	5.798	27.52	758.54
SHR CuNP_6.5_		2.325 ^a^	5.386	36.42	975.24
SEM		0.037	0.071	0.112	0.416
Blood pressure (R)					
	WKY	2.362	1.693 ^a^	33.07 ^a^	557.68 ^b^
	SHR	2.353	1.352 ^b^	22.54 ^b^	615.73 ^a^
Cu source (C)					
	CuCO_3 6.5_	2.145	0.516^c^	14.98 ^c^	580.26 ^b^
	CuCO_3 3.25_ + CuNP_3.25_	2.570	2.529^b^	40.63 ^a^	593.15 ^b^
	CuNP_6.5_	2.289	5.592^a^	31.97 ^b^	866.89 ^a^
*p* value					
R effect		0.384	0.031	0.012	0.047
C effect		0.008	<0.001	<0.001	0.008
R × C interaction		0.044	0.749	0.052	0.174

^1^ WKY CuCO_3 6.5_—normotensive rats fed 6.5 mg/kg copper carbonate salt; SHR CuCO_3 6.5_—hypertensive rats fed 6.5 mg/kg copper carbonate salt; WKY CuCO_3 3.25_ + CuNP_3.25_—normotensive rats fed 3.25 mg/kg CuNPs plus 3.25 mg/kg copper carbonate salt; SHR CuCO_3 3.25_ + CuNP_3.25_—hypertensive rats fed 3.25 mg/kg CuNPs plus 3.25 mg/kg copper carbonate salt; WKY CuNP_6.5_—normotensive rats fed 6.5 mg/kg CuNPs; SHR CuNP_6.5_—hypertensive rats fed 6.5 mg/kg CuNPs. ^a,b,c^ Means within a column with different superscript letters are significantly different (*p* < 0.05); differences between groups (WKY CuCO36.5, SHR CuCO_3 6.5_, WKY CuCO_3 3.25_ + CuNP _3.25_, SHR CuCO_3 3.25_ + CuNP_3.25_, WKY CuNP_6.5_, SHR CuNP_6.5_) are indicated with superscripts only in the case of a statistically significant R × C interaction (*p* < 0.05). SEM—pooled standard error of mean (standard deviation for all rats divided by the square root of the number of rats, *n* = 60); MDA—malondialdehyde; GSH + GSSG—total glutathione; SOD—superoxide dismutase; CAT—catalase.

**Table 5:** Redox status in small intestinal tissue.

Dietary Treatment ^1^		MDA µmol/g	GSH + GSG umol/kg	SOD U/g	CAT U/g
WKY CuCO_3 6.5_		5.603 ^a^	19.564	11.068	1892.28 ^a^
SHR CuCO_3 6.5_		4.135 ^b^	7.037	11.163	1700.45 ^b^
WKY CuCO_3 3.25_ + CuNP_3.25_		6.429 ^a^	10.811	10.618	1263.13 ^d^
SHR CuCO_3 3.25_ + CuNP_3.25_		6.855 ^a^	18.109	10.551	1478.23 ^c^
WKY CuNP_6.5_		4.988 ^ab^	8.679	11.362	1756.64 ^b^
SHR CuNP_6.5_		5.075 ^ab^	8.552	10.769	1443.23 ^c^
SEM		0.026	0.044	0.124	0.892
Blood pressure (R)					
	WKY	6.016	15.188	10.843	1577.71
	SHR	5.495	12.573	10.857	1589.34
Cu source (C)					
	CuCO_3 6.5_	4.869	13.301 ^a^	11.115	1796.37
	CuCO_3 3.25_ + CuNP_3.25_	6.642	14.460 ^a^	10.584	1370.68
	CuNP_6.5_	5.032	8.616 ^b^	11.065	1599.93
*p* value					
R effect		0.072	0.477	0.647	0.824
C effect		0.048	<0.001	0.068	0.006
R × C interaction		0.049	0.062	0.077	0.004

^1^ WKY CuCO_3 6.5_—normotensive rats fed 6.5 mg/kg copper carbonate salt; SHR CuCO_3 6.5_—hypertensive rats fed 6.5 mg/kg copper carbonate salt; WKY CuCO_3 3.25_ + CuNP_3.25_—normotensive rats fed 3.25 mg/kg CuNPs plus 3.25 mg/kg copper carbonate salt; SHR CuCO_3 3.25_ + CuNP_3.25_—hypertensive rats fed 3.25 mg/kg CuNPs plus 3.25 mg/kg copper carbonate salt; WKY CuNP_6.5_—normotensive rats fed 6.5 mg/kg CuNPs; SHR CuNP_6.5_—hypertensive rats fed 6.5 mg/kg CuNPs. ^a,b,c,d^ Means within a column with different superscript letters are significantly different (*p* < 0.05); differences between groups (WKY CuCO_3 6.5_, SHR CuCO_3 6.5_, WKY CuCO_3 3.25_ + CuNP _3.25_, SHR CuCO_3 3.25_ + CuNP_3.25_, WKY CuNP_6.5_, SHR CuNP_6.5_) are indicated with superscripts only in the case of a statistically significant R × C interaction (*p* < 0.05). SEM—pooled standard error of mean (standard deviation for all rats divided by the square root of the number of rats, *n* = 60); MDA—malondialdehyde; GSH + GSSG—total glutathione; SOD—superoxide dismutase; CAT—catalase.

## Data Availability

Not applicable.
